# Electrochemical Properties of Carbon Fibers from Felts †

**DOI:** 10.3390/molecules27196584

**Published:** 2022-10-04

**Authors:** Guilhem Pignol, Patricia Bassil, Jean-Marie Fontmorin, Didier Floner, Florence Geneste, Philippe Hapiot

**Affiliations:** 1 Univ. Rennes, CNRS, ISCR—UMR 6226, F-35000 Rennes, France; 2 Kemiwatt, 11 Allée de Beaulieu, CS 50837, CEDEX 7, F-35708 Rennes, France

**Keywords:** carbon fiber, microelectrode, cylindrical diffusion, electron transfer kinetics

## Abstract

Electrochemical behaviors of individual carbon fibers coming from carbon felts were investigated using two different redox couples, 1,1′-dimethanolferrocene and potassium ferrocyanide. Electrochemical responses were examined after different oxidation treatments, then simulated and interpreted using the Kissa 1D software and existing models. Our experiments indicate that a crude carbon fiber behaves as an assembly of sites with different electrochemical reactivities. In such case, the Butler–Volmer law is not appropriate to describe the electron transfer kinetics because of the large created overpotential. Oxidation of the fiber erases the effect by increasing the kinetics of the electron transfer probably by a homogenization and increase of the reactivity on all the fiber. Additionally, analysis of the signal shows the large influence of the convection that affects the electrochemical response even at moderate scan rates (typically below 0.1–0.2 V s^−1^).

## 1. Introduction

Carbon electrode materials are used in numerous applications notably in redox flow batteries where electrodes are often composed of carbon felts [1]. The morphologies of the fibers in the felt as well as their surface functionalization are the key parameters to understand the behaviors of the electrode [2]. For example, it is well-known that the oxidation of a carbon electrode considerably modifies its electrochemical characteristics [2] providing an empirical way for improving the current density or overpotential [3]. Studying directly a carbon felt is complicated both because it is composed of a variety of fibers and of a three-dimensional porous geometry. To circumvent these difficulties, a useful suggestion was to extract a single fiber from the felt and to study its electrochemical behavior as a model of the felt itself and in the final purpose of an optimization of the electrochemical properties [4,5,6]. This approach allows a large simplification of the simulations with providing valuable information on the electrochemical processes. In these works, examinations of the single fiber mainly rely on impedance spectroscopy and then extended to the felt itself. This approach was recently completed using cyclic voltammetry in combination with numerical simulations to characterize the carbon fiber but in a limited scan rate range [6]. The rigorous treatment of the system is not obvious as a fiber behaves as a cylinder electrode [7] with an electrochemical reactivity that is generally not homogeneous on its surface [8,9]. This question is in direct relation with the old problem (but still concerning active research) of the partially blocked electrode that was notably examined by C. Amatore et al. for a planar electrode [10] or later by R.G. Compton et al. for an array of microelectrodes [11]. Additionally, other phenomena such as the natural convection are likely affecting the electrochemical response of a cylinder electrode [12,13].

In the present work, we have re-examined the electrochemical behavior of an individual carbon fiber extracted from a felt by cyclic voltammetry performed at different scan rates . We used two common redox couples as electrochemical probes, the one-electron oxidation of 1,1′-dimethanolferrocene and the one-electron oxidation of potassium ferrocyanide [K_4_Fe(CN)_6_]. These two redox couples were chosen as they present different interactions with a carbon surface [2,14]. The oxidation of ferrocene is almost unsensitive to the chemical nature of carbon surface contrarily to ferrocyanide that requires a close interaction between the molecule and the carbon materials for an efficient electron transfer [2]. The electrochemical responses were examined before and after different oxidations of the fiber, then the curves were simulated and interpreted using numerical simulations based on theoretical models for a cylinder electrode [7,15] and the help of the KISSA 1D software [16]. The possibilities and errors/complications identified from the simulations have then been evaluated for the use of these fibers as quantitative sensors, for example for applications in redox flow batteries where the measurement of concentrations of the redox species is a way of estimating the state of charge.

## 2. Experimental Section

### 2.1. Chemicals

All chemicals were commercially available and used in their analytical grade. All solutions were made using ultrapure water (Purelab Classic UV from Veolia, France) 18.2 MΩ cm at 25 °C). 1,1′-dimethanolferrocene was from Alfa Aesar (Thermo Scientific Chemicals, Karlsruhe, Germany) (99% purity). Potassium ferrocyanide was from Acros Organics (Thermo Scientific Chemicals, Karlsruhe, Germany) (99% purity). Two solutions were considered: 1,1′-dimethanolferrocene in 0.1 mol L^−1^ H_2_SO_4_ and 0.5 mol L^−1^ KCl, potassium ferrocyanide in 0.5 mol L^−1^ KCl.

**Figure 1:**
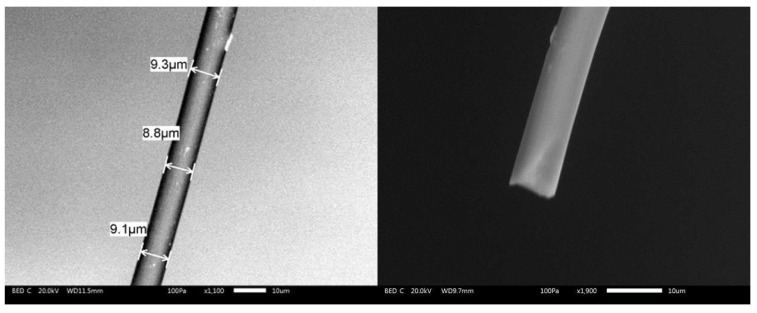
Typical SEM images of two carbon fibers used in this study (JSM-IT300 SEM).

Fibers were extracted from the same batch of carbon felts (Pan-Based type Carbon Fiber, SGL carbon SE) with a purpose of comparison and immobilized in a glass tube following an adaption of a published method [17]. The contact between a copper wire and the fiber was made using conductive silver and the sealed with a pipette tip and cyanoacrylate glue. It provides a sufficiently robust setup for making several experiments with the same fiber. Their length was approximately adjusted to 1 mm but may sightly vary between samples. Different fibers were studied: crude fibers that were only rinsed with water and ethanol after their extraction from the carbon felt (type 1), fibers that were electrochemically oxidized in the cell by applying a 2 V potential for 2 min (type 2), and fibers that were strongly oxidized (type 3). These fibers (type 3) were extracted from an oxidized carbon felt which have undergone an electrolysis at pH 2 by applying a constant current of 1 A during 90 min [18]. Carbon fibers were optically examined by scanning electron microscopy (SEM) to determine their radius that was estimated as 4.5 µm (see Figure 1).

### 2.2. Electrochemical Procedures

A standard three-electrode configuration was employed for all electrochemical measurements, using an Autolab PGSTAT30 (Metrohm, Villebon-sur-Yvette, France). The working electrode was the carbon fiber. The counter electrode was a platinum wire and the reference electrode was KCl saturated Ag/AgCl. Dissolved oxygen was purged by bubbling argon before all experiments and an inert atmosphere was maintained during experiments. For each fiber and scan rate, the applied potential was limited to a range between −0.4 and 1 V to avoid any further oxidation of the fiber during the voltammetry experiments.

Numerical simulations were performed with the Kissa 1D software package [16] using the default parameters of the calculations.

## 3. Results and Discussion

### 3.1. Characterization of the Fiber with 1,1′-Dimethanolferrocene

Two different redox couples were chosen as probes because of their different electrochemical response on carbon materials. Ferrocene derivatives (Fc) are known for presenting a fast electron transfer that is almost unsensitive to the chemical nature of the carbon surface and thus are well adapted to characterize a mass transfer process of the fiber [2].

**Figure 2:**
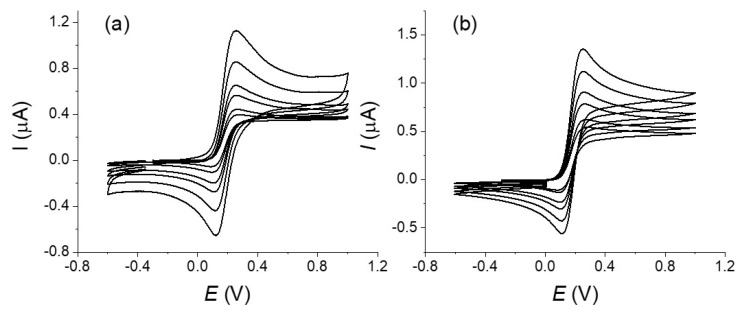
Voltammograms of a 5 × 10^−3^ mol L^−1^ of a 1,1′-dimethanolferrocene solution in H_2_SO_4_ 0.1 mol L^−1^ and KCl 0.5 mol L^−1^ (WE: carbon fiber; CE: platinum wire; RE: Ag/AgCl 3M) at different scan rate: 10, 25, 100, 200, 500 and 1000 mV s^−1^. (**a**) Crude fiber (type 1), (**b**) after strong oxidation treatment (type 3).

**Figure 3:**
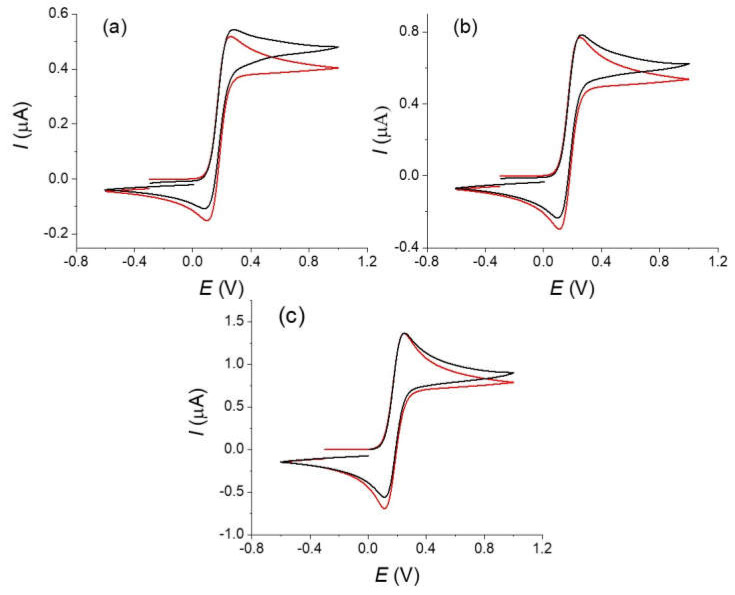
Simulation (red) and experimental curves (black) recorded on an oxidized fiber (type 3) of a 5.10^−3^ mol L^−1^ solution of 1,1′-dimethanolferrocene in 0.1 mol L^−1^ H_2_SO_4_ and 0.5 mol L^−1^ KCl. Scan rates ν = (**a**) 0.01, (**b**) 0.1, (**c**) 1 V s^−1^. Parameters of the simulations; *r* = 4.5 µm; *l* = 0.9 mm; α = 0.5; *E°* = 0.175 V; *k_s_* = 0.025 s^−1^.

Figure 2 and Figure 3 show the voltammograms recorded for a solution of Fc on a carbon fiber before (type 1) and after its strong oxidation (type 3) at different scan rates. Reversible voltammograms are observed for all scan rates and for all the tested fibers. No considerable effect of the oxidation on the electrochemical response of Fc is noticeable between a fiber that was simply rinsed by water and ethanol and a fiber after its oxidation. This is in agreement with the expected behavior for such redox couple and shows that Fc is well adapted for a characterization of the mass transport at the carbon fiber (see for example reference [19]). To go in more details, we performed a series of simulations of the curves considering a simple mechanism with quasi-reversible electron transfer under cylindrical diffusion [7]. The analytical treatment of the quasi-reversible electron transfer provides useful solutions in the limiting cases and/or approximate solutions for practical situations and the parameters that control the system. The electrochemical response depends on two dimensionless parameters: (i) a geometrical parameter that characterizes the cylindrical factor

(1) $$\beta =2{\left(\frac{\mathrm{DRT}}{{F\nu r}^{2}}\right)}^{1/2}$$

(ii) a dimensionless charge transfer rate constant that characterized the reversibility of the transfer

(2) $$\Lambda =k_{s}{\left(\frac{RT}{F\nu D}\right)}^{1/2}$$

*D* is the diffusion constant, *R* is the perfect gas constant, *T* the absolute temperature, *F* is the faradaic constant, *v* is the scan rate, *r* the radius of the cylinder, *k_s_* is the standard heterogenous charge transfer rate constant. When *β* tends to zero, the diffusion approaches a linear behavior and when *L* tends to infinite, the system becomes totally reversible and the behavior is just controlled by the diffusion [7].

The radius of the cylinder of our fiber was estimated as 4.5 µm according to the SEM experiments and this value was used in simulations. Considering a diffusion coefficient *D* = 6 × 10^−6^ mol cm^−2^ [20] and scan rates in the range 0.1–1 V s^−1^, this leads to *β* values in the range of 5.5–1.7 that corresponds to intermediate situation between linear and pure cylindrical diffusion conditions, the highest scan rate being the closest to the linear diffusion conditions [7]. For the simulations of the voltammograms and for simplicity of the treatment, we used the Kissa 1D software that provides an accurate simulation of electrochemical processes under cylindrical electrode and is well-adapted for calculating the voltammograms [16]. We considered the Butler–Volmer law for describing the kinetics of charge transfer at the electrode with a transfer coefficient *α* = 0.5 [21]. For a relatively fast system as we have here, this value has little influence on the final response [20]. The length of the fiber, E° and *k_s_* values were then adjusted in order to fit the peak current and the peak-to-peak potentials difference Δ*Ep* at a scan rate of 1 V s^−1^ and were kept the same for all curves. This scan rate was chosen as a compromise to limit the effect of the convection (see below) and possible artifacts from the ohmic drop (see for example [20] and references therein). A good agreement between experimental and theoretical voltammograms was obtained considering values of *E°* of 0.175 V and a standard electron transfer rate constant *k_s_* = 0.025 s^−1^ for the 1,1′-dimethanolferrocene/1,1′-dimethanolferrocenium couple that agrees with values reported in the literature for similar molecules in water. However, we could consider this value as a low limit as we cannot guarantee the total absence of a residual ohmic that would make the apparent kinetics of the electron transfer looking lower [20]. If the results between simulation and experimental curves present a good agreement, differences appear on the evolution of the current after the oxidation peak notably at the lower simulated scan rates. We could also notice that the voltammograms recorded at the lowest scan rate (see Figure 2) present a totally S-shape character which is not possible if only considering a cylindrical diffusion with our electrode characteristics. Similar tendency is visible in the voltammograms published in the literature for comparable redox systems [6,8]. This indicates that the experimental diffusion regime differs from the simulation at the lowest scan rate, the more pronounced S-Shape character suggesting the interference of convection on the recorded voltammograms even at the highest scan rates [12].

### 3.2. Oxidation of Ferrocyanide on the Carbon Fiber

Contrarily to ferrocene oxidation, electron transfer rate of the oxidation of [Fe(CN)_6_]^4−^ depends on the nature of the carbon electrode surface and more generally on the carbon materials [2]. Several studies were made to determine the electron transfer rate on these surfaces notably to rationalize how the surface state could affect the kinetic [2]. Even if some aspects remain unclear, it is admitted that the presence of oxidative sites could considerably increase the kinetics of the electron transfer [10,15,22].

**Figure 4:**
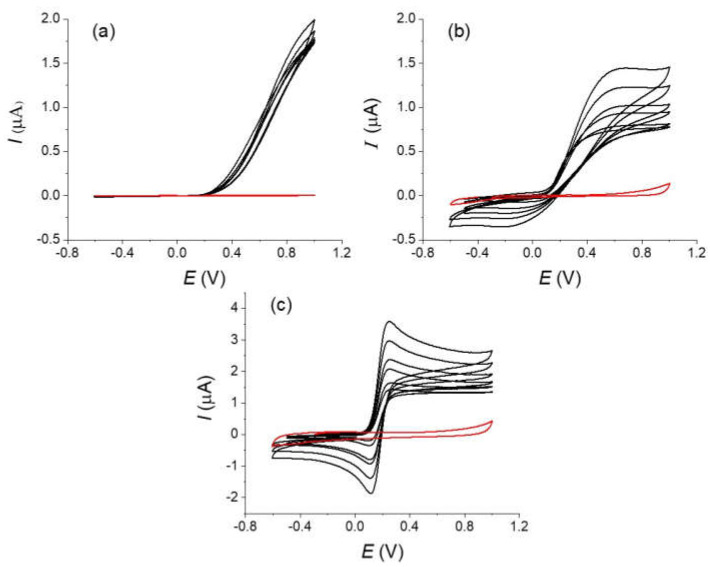
Voltammograms of a 10^−2^ mol L^−1^ of a potassium ferrocyanide solution in KCl 0.5 mol L^−1^ at different scan rates (black lines) : 0.01, 0.025, 0.1, 0.2, 0.5, and 1 V s^−1^ (**a**) without treatment, (**b**) same fiber after an in-situ oxidation (type 2), and (**c**) fibers after ex situ treatment (type 3). Red lines are the background currents measured at the highest scan rate (1 V s^−1^).

Typical experimental voltammograms of the oxidation of the ferrocyanide are shown on Figure 4. Voltammograms were recorded on similar carbon fibers but with different types of treatment: for an original non-treated fiber that was simply cleaned with water, a carbon fiber that was electrochemically oxidized in the voltammetry cell and finally a fiber that strongly oxidized (see experimental part).

**Figure 5:**
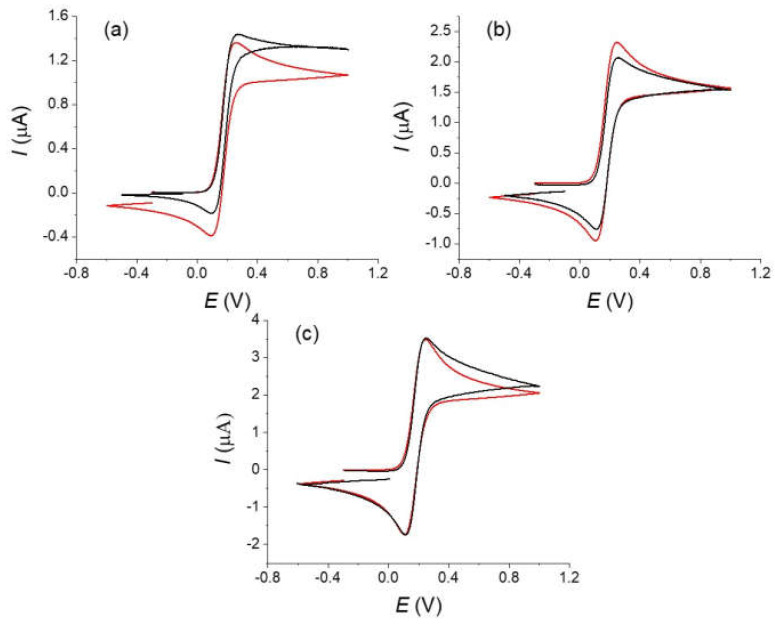
Simulations and experimental curves recorded on fully oxidized fibers (type 3) of a 1.10^−2^ mol L^−1^ solution of potassium ferrocyanide in 0.5 mol L^−1^ KCl at different scan rates : (**a**) 0.01, (**b**) 0.1, (**c**) 1 V s^−1^. Parameters of the simulations are: *r* = 4.5 µm; *l* = 1.05 mm; *α* = 0.5; *E°* = 0.17 V; *k_s_* = 0.025 s^−1^. Black lines are the experimental curves. Red lines are the simulations.

A simple examination of the different voltammograms shows the considerable effects of the oxidation treatment on the obtained response in agreement with reports in the literature [2,4,6]. Notice that we found large variations with fibers simply rinsed with water (type 1), which could be explained by a non-defined oxidation state. The voltammograms obtained with the strongly oxidized fiber (type 3) are reversible with a low peak-to-peak potential difference in opposition to the fiber without treatment (type 1) for which a totally irreversible electron transfer with a large overpotential is observed. The curve for the fiber with the moderate oxidation (type 2) appears as an intermediate situation. The background currents measured on the same fibers in a blank solution slightly increase with the oxidation treatment but remain negligible in the global current and will not be considered in the following simulations (see the red curves on Figure 5).

In the first approach, we limit the simulations using the cylindrical diffusion conditions with the KISSA 1D software of the voltammograms recorded on the fully oxidized fiber (type 3). We just considered a simple electron transfer described by the Butler–Volmer kinetics law as for the previous with the oxidation of Fc. A good agreement was obtained for the highest scan rate using an electron transfer standard rate constant *k_s_* around 0.025 cm s^−1^, a value that falls in line with what is obtained for the oxidation of ferrocyanide on glassy carbon electrodes in comparable experimental conditions [20]. As observed above for the oxidation of Fc, the current after the peak differs from the simulation and presents a more “plateau shape” (see discussion below).

**Figure 6:**
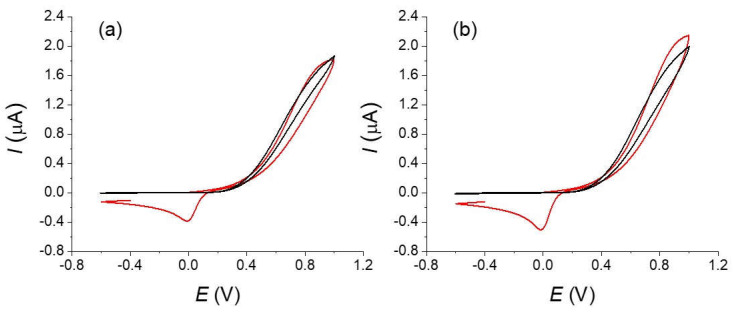
Simulation (in red) and experimental curves (in black) on the crude carbon fiber of a 10^−2^ mol L^−1^ potassium ferrocyanide solution in 0.5 mol L^−1^ KCl water. Scan rate (**a**) 0.5, (**b**) 1 V s^−1^. Parameters used for the simulation are *r* = 4.5 µm; *l* = 1.1 mm; *α* = 0.82, *E°* = 0.17 V, *k_s_* = 1.5 × 10^−4^ s^−1^. Black lines are the experimental curves. Red lines are the simulations.

Similar conditions and a simple electron transfer described with a Butler–Volmer law were then considered to simulate the voltammograms recorded with the untreated fiber (type 1) (see Figure 6). A reasonable agreement was obtained for the oxidation current with *k_s_* value around 1.5 × 10^−4^ cm s^−1^ that is more than two orders smaller than the *k_s_* measured on the chemically oxidized fiber for the same couple. C. Amatore et al. have provided a very useful model for describing a partially blocked electrode [6]. This model in its simplified form predicts that for a partially blocked electrode the apparent electron transfer kinetics is affected by the following formula

(3) $$k_{S}^{ap}=k_{S,0\;}^{ap}\left(1-\theta \right)$$

where θ is the fractional coverage of the electrode by the blocking film. Using this approximation means that less than one percent of the fiber is really efficient for the oxidation of [Fe(CN)_6_]^4−^ that obviously justifies the treatment of the fiber before its use in an electrochemical application.

**Figure 7:**
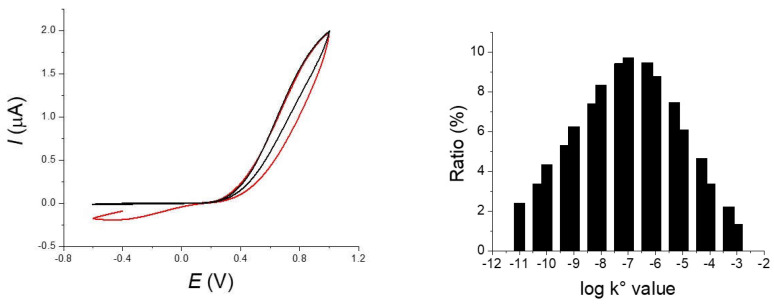
Simulation (red) and experimental curves (black) of a 10^−2^ mol L^−1^ solution of potassium ferrocyanide in 0.5 mol L^−1^ KCl water solution at 1 V s^−1^. Parameters used for the simulation are r = 4.5 µm; *l* = 1.05 mm; *α* = 0.5; *E°* = 0.175 V and a distribution of values for *k_s_* showed in the histogram.

However, a simple electron transfer does not totally account for the observed results. First to reproduce the slope of the current with the potential, we need to introduce in the simulation a value of the transfer coefficient *α* around 0.8 that is considerably different from the 0.5 of the Butler–Volmer Law. Second, we do not observe a return current (reduction) in our experiments contrarily to the prediction of the simulation. The oxidation of [Fe(CN)_6_]^4−^ occurs with a large overpotential on the fiber (0.7–0.8 V above the *E°*) and a quadratic activation-free energy relation as in the Marcus model is probably more appropriate for describing the electron transfer kinetics. This could explain that the apparent transfer coefficient is largely different from 0.5. Indeed, in the framework of the Marcus model, *α* is defined as

(4) $$\alpha =0.5\;\left(1+\frac{E-E{}^{\circ}}{4∆G_{o}^{*}}\right)$$

where $∆G_{o}^{*}$ is the intrinsic barrier, leading to values of *α* significantly different from 0.5 in case of large overpotential [21]. In the models relative to a blocked electrode, all sites were considered equivalent with a similar reactivity [10]. This approximation is probably not valid in our case for the crude carbon fiber (type 1). The problem of the non-equivalent reactive sites, for example reactive sites displaying different charge transfer rates and sizes, is much more complicated than the blocked electrode (or microdisks array) under Nernstian conditions treated above (for a full discussion about that point, see for the reference) [23]. For the best of our knowledge, this has not been totally solved today but this is an active subject in theoretical electrochemistry. To provide a simplified representation of our experimental situation, we simply considered an electrochemical system composed of a collection of reactive sites but with different electron transfer constant *k_s_*. Basically, it corresponds to a situation of non-interacting sites with different electrochemical reactivities. We used a gaussian distribution of the rate constants that was approximated by a normalized sum of currents for a discrete series of *k_s_*. The obtained simulations are represented on Figure 7 with the distribution of *k_s_*. As observed, a good fitting was obtained with our experimental data for the highest scan rate even when using a transfer coefficient close to 0.5.

### 3.3. Influence of the Convection on the Electrochemical Response

**Figure 8:**
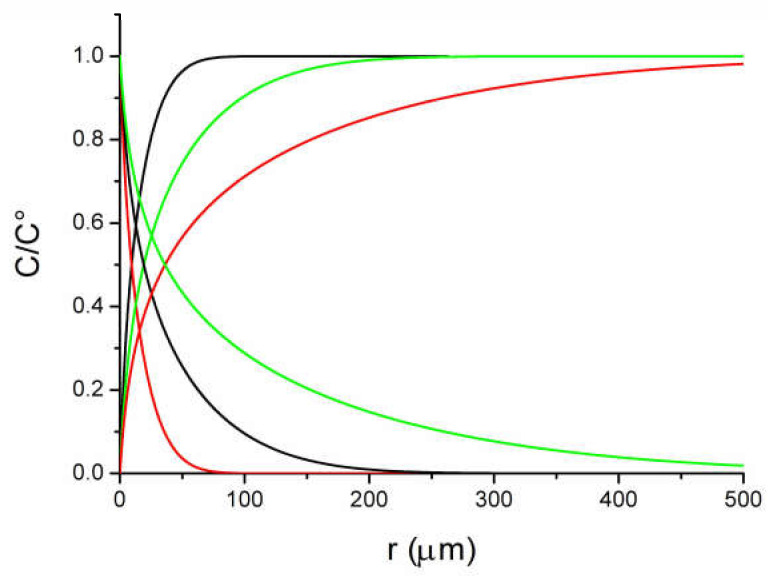
Concentration profiles at a 4.5 mm radius fiber with the parameter of Figure 3. Scan rates: 0.01 (green), 0.1 (black), 1 (red) V s^−1^ calculated with Kissa 1D.

Using a single carbon fiber as an amperometric sensor is a simple concept that could be envisaged to measure the concentration of a redox species as a simple low-cost and miniaturized device notably in redox flow batteries [6]. It would present the advantage that the sensor is made of the same material than the main electrodes of the battery. Additionally, even a micrometric radius fiber with a 1 mm length provides a sufficiently large current for an easy measurement with a good signal/noise ratio. In a convenient procedure, one would expect using the fiber at a reasonably long measurement to simplify the measurement setup and detection. As noticed above, the voltammograms recorded at scan rate at 0.1 V s^−1^ and below for the oxidation of Fc (Figure 2) or the oxidation of [Fe(CN)_6_]^4−^ on an activated fiber (Figure 4), the current after the peak shows a “plateau” shape feature meaning that the current is higher than expected. As discussed before, despite similar micrometric sizes between a fiber electrode and a disk microelectrode, the extension of the diffusion layer for a cylindrical electrode has an extension comparable to that observed for linear diffusion [7]. This situation is totally different from the diffusion layer for spherical symmetry electrode that are in the order of few times the radius of the electrode. It results that an electrode with a cylindrical symmetry is more sensitive to “natural” convection than a disk microelectrode with a similar radius. This raises concerns about the effect of convection and the possibility of using current measurements to evaluate a concentration with a fiber. In a first approximation, the effect of the convection is to limit the extension of the diffusion layer making the voltammogram with a more plateau like shape (S-Shape) [10,12]. Taking into account the effects of the “natural” convection on the response of a cylindrical fiber is not rigorously simple. Indeed, the “natural” convection layer presents a different symmetry than the cylindrical diffusion layer and the calculation requires a more precise description [12]. However, the general influence of the convection to limit the extension of the diffusion layer remains. An evaluation of the convection effect could be obtained by looking at the diffusion concentration profiles and the extension of the layer. Concentration profiles were calculated with Kissa 1D (see Figure 8) at the level of the inversion potential of the voltammogram, at different scan rates and with the same geometrical parameters of Figure 3 (a 4.5 μm radius fiber, scan rates of 0.01, 0.1 and 1 V s^−1^). Considering a reasonable thickness for the natural convection layer around 100 μm, one could see that the extension of the diffusion layer will already be affected at scan rates below 0.1 V s^−1^ and that a voltammogram recorded at 0.01 V s^−1^ will be almost controlled by the natural convection. If we now consider a convection layer of 50 μm, even a scan rate of 1 V s^−1^ will be disturbed. Looking at the experiments of Figure 3 for Fc or Figure 5 for [Fe(CN)_6_]^4−^ oxidations on type 3 fiber, occurrence of convection explains that the voltammograms display a plateau-shape character at the lower scan rates. We could also derive a sort of empirical value around 50–100 μm for the convection layer thickness for a stationary solution.

**Figure 9:**
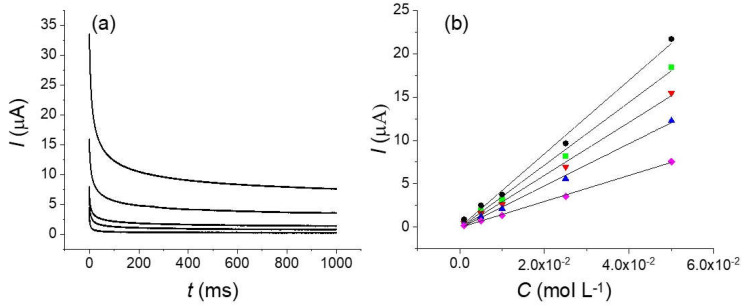
(**a**) Chronoamperogram obtained for different concentrations of potassium ferrocyanide from top to bottom: 5 × 10^−2^, 2.5 × 10^−2^, 1 × 10^−2^, 5 × 10^−3^ and 1 × 10^−3^ mol L^−1^. (**b**) Current vs. concentration at different measurement times 10 (black) , 20 (green), 40 (red), 100 (blue), 1000 (pink) ms. Applied potential 0.65 V.

Because it is difficult to quantify the possible unfavorable evolution of the natural convection with the environmental conditions, we prepared some test solutions of K_4_[Fe(CN)_6_] at different concentrations in KOH 0.1 mol L^−1^. We applied a constant potential at 0.65 V and measured the current as function of time. We used an oxidized fiber to ensure that the system is controlled by the mass transfer with negligible influence of the electron transfer kinetics. Variations of the current at different concentrations and measurement times (10^−2^ to 1 s) are shown on Figure 9. As observed on Figure 9a, well-defined signals are obtained with a good ratio signal/noise. However, the linearity of the concentration/current decreases for measurement times below 40 ms because of the presence of the background/capacitance current of the oxidized fiber and the limited response time of our electrochemical setup.

**Figure 10:**
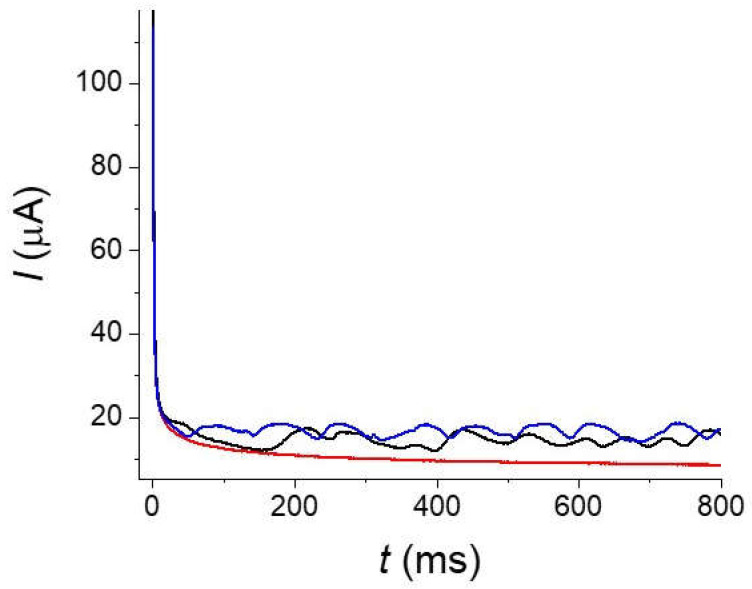
Chronoamperogram of a 10^−1^ mol L^−1^ solution of potassium ferrocyanide at pH 13 in KOH under different stirring. Stationary solution (red), 200 RPM (black), 600 RPM (blue).

For the longer times, a good linearity was obtained. According to the previous estimation, measurement time of 1 s corresponds approximatively to a voltammogram of 1 V s^−1^ and we have seen that this is the limit for neglecting the convection in a stationary solution. A simple experimental test consists in stirring the solution to modify the convection around the fiber. As seen on the recorded chronoamperogram of Figure 10, even with a low stirring, the current is strongly disturbed and multiplied by a ratio higher than 2–3 at the longest times. For envisaging the use of the fiber in the conditions where the convection is negligible, we need measurement times no longer than 20 ms (below 10 ms will be better). These two constraints, on the one hand, the short times and occurrence of background currents associated to bandpass limitations and on the other hand, the influence of the convection at long time, strongly limit the interest of using a fiber for measuring concentrations but this will probably be possible with an adequate electronic setup.

## 4. Conclusions

In conclusion, a carbon fiber that may be seen as a simple object displays in fact a complicated response for which a full description remains challenging. This is notably due to their inhomogeneous properties and the large interference of convection that is already present in most common conditions of use.

Our experiments suggest that a crude carbon fiber behaves as an assembly of sites with different electrochemical reactivities. In such a case, Butler–Volmer law is not appropriate to describe the electron transfer kinetics because of the large created overpotential. Oxidation of the fiber erases the effect by increasing the kinetics of the electron transfer, probably by homogenization of the reactivity on all the fiber. This is accompanied by a light increase in the background current that could limit the use of the fiber in a short time.

Concerning the use of a carbon fiber as a sensor, such application needs short measurement times that would require an improvement in the electrode in terms of background current and the use of a fast potentiostat. Using long (or even moderately long) time for the measurement is clearly not possible because of the cylinder geometry. In such conditions, the electrochemical response is affected by the natural convection and thus is too sensitive to the environment for a practical use.

## Data Availability

The data presented in this study are available upon request from the corresponding author.
